# Biological Activities of In-House Developed *Haloxylon griffithii* Plant Extract Formulations

**DOI:** 10.3390/plants10071427

**Published:** 2021-07-13

**Authors:** Shagufta Kamal, Ismat Bibi, Kanwal Rehman, Ameer Fawad Zahoor, Amna Kamal, Fatima Aslam, Fatmah Ali Alasmary, Tahani Mazyad Almutairi, Hassna Mohammed Alhajri, Siham A. Alissa, Hafiz M. N. Iqbal

**Affiliations:** 1 Department of Biochemistry, Government College University, Faisalabad 38000, Pakistan; Fateemahchaudhary@gmail.com; 2 Department of Chemistry, The Islamia University of Bahawalpur, Bahawalpur 63100, Pakistan; drismat@iub.edu.pk; 3 Department of Pharmacy, University of Agriculture, Faisalabad 38000, Pakistan; kanwal.akash@gmail.com; 4 Department of Chemistry, Govt. College University, Faisalabad 38000, Pakistan; fawad.zahoor@gmail.com; 5 Department of Chemistry, University of Agriculture, Faisalabad 38000, Pakistan; amina89@gmail.com; 6 Chemistry Department, College of Science, King Saud University, P.O. Box 2455, Riyadh 11451, Saudi Arabia; fasmari@ksu.edu.sa (F.A.A.); talmutari1@ksu.edu.sa (T.M.A.); 441203417@student.ksu.edu.sa (H.M.A.); 7 Department of Chemistry, College of Science, Princess Nourah bint Abdulrahman University, Riyadh 11671, Saudi Arabia; saalissa@pnu.edu.sa; 8 Tecnologico de Monterrey, School of Engineering and Sciences, Monterrey 64849, Mexico

**Keywords:** plant extract, antimicrobials, phytoconstituents, time kill assay, haemolytic assay, phytochemicals, thrombolytic activity

## Abstract

The therapeutic potential of whitish glaucous sub-shrub *Haloxylon griffithii* (*H*. *griffithii*), abundantly present in southern regions of South Asia, has been neglected. The current study aimed to assess the phytochemicals and pharmacological potential of native and gemm forms of *H*. *griffithii*. Results of antimicrobial activity revealed that all tested bacteria were susceptible at concentrations ≤50 µg/mL, while tested fungal species were susceptible at ≤25 µg/mL. The values of minimum bactericidal concentrations (MBCs) ranged between 10.75 ± 0.20 to 44.25 ± 0.42 µg/mL, 8.25 ± 0.02 to 28.20 ± 0.80 µg/mL. The value of minimum inhibitory concentration (MIC) of all microbial species was ≤100 µg/mL and the antibiotic mechanism showed that both extracts were highly bactericidal and fungicidal. Results of average log reduction of viable cell count in time kill assay indicated that *Pseudomonas aeruginosa* (*P*. *aeruginosa*) NCTC 1662, *Candida albicans* (*C*. *albicans*) IBL-01, *Candida* *krusei* (*C*. *krusei*) ATCC 6258, and *Aspergillus flavus* (*A*. *flavus*) QC 6158 were the most susceptible microbial species. High performance liquid chromatography (HPLC)-based quantification confirmed the presence of gallic acid p.coumeric acid catechin, vanillin, ellagic acid, and salicylic acid, while in native extract only gallic acid. Native and gemm extracts exhibited excellent radical scavenging potential measured by 1,1-diphenyl-2-picryl-hydrazyl radical scavenging assay. Significant thrombolytic activity was found in both extracts with negligible haemolytic activity. Highest percent (%) clot lysis was observed with gemm extracts (87.9 ± 0.85% clot lysis). In summary, we infer that valuable evidence congregated can be exploited for better understanding of gemm *H*. *griffithii’s* health benefits, further, to increase its utility with enriching dietary sources of health-promoting compounds.

## 1. Introduction

Antibiotic resistance explicates pharmacological therapy and epidemiology of contagious diseases in medical jargon; however, the escalating trend of antimicrobial resistance is a rising apprehension of modern pharmaceutical arena [1]. Extensive efforts have been focused to discover novel antimicrobial agents of plants or microbial origin to prevent the dangers of pathogenic-resistant antimicrobial agents [2,3,4,5,6]. Plants, being rich in numerous pharmacologically active compounds, are the natural blueprints to overcome amplified global health challenges. Around 70% of modern pharmaceutical products are made up of plants or their active ingredients due to their supreme chemical diversity [7].

Gemmotherapy is a relatively new phytotherapy technique. Gemmotherapy is a super active type of herbal medicine consisting of many important enzymes and vitamins that are liberated at the time of high growth [8,9]. In view of gemmotherapy, no gemm formulation of *H*. *griffithii* has yet been commenced, however, some gemm formulations have been explored as part of herbal medicines for homeo treatments. *H*. *griffithii* is a subshrub that is dispersed in the southern areas of Pakistan and a member of Chenopodiaceae family, known as Cat tail species, consisting of 1200 species with 100 genera [10]. *H*. *griffithii* is one of the anti-infective agents that is reported to be used as a cure for diabetes, eye-disorders, and also act as an anti-inflammatory and antiseptic agent [11].

A phytopharmacological approach should not be based on initial selection of plants, as it is evident that crude extracts show biological activity [12]. Although biologically active substances such as glycosides, tannins and saponins are found to be distributed in the Chenopodiaceae family, these plants are not well used due to a lack of knowledge and technique. The phyto-constituents, including alkaloids, glycosides, flavonoids and saponins, are considered to be the principal antibiotics of *H*. *griffithii* because these phyto-constituents are the defensive mechanism against various pathogens, while various minerals are also known to possess medicinal properties [13]. Antioxidant and antimicrobial activities of *H*. *griffithii* have not been explored yet because of its presence in the southern regions of Pakistan. The present study was designed to check the comparative effectiveness in the treatment of bacterial or fungal infections, antioxidant potential, phytoconstituents, mineral profile, and thrombolytic and cytotoxic potential of both native and gemm extract of *H*. *griffithii*.

## 2. Materials and Methods

### 2.1. Collection of Plant Material and Sample Preparation

Fresh plants of *Haloxylon griffithii* were obtained from the Baluchistan southern region of Pakistan and its inborn form was also bought from the southern region of Pakistan and identified by Dr. Muhammad Azeem, Dept. of Botany, Faculty of Life Sciences, Govt. College University, Faisalabad. The shade-dried voucher specimen was deposited in the Herbarium of medicinal plants, Govt. College University, Faisalabad under herbarium number 205-bot-2018.

Both fresh growing parts (fresh growing shoots, roots and leaves) of *Haloxylon griffithii* and mature parts (shoots, leaves and roots) of *H. griffithii* were used. Both plant materials (immature and mature) were washed thoroughly with distilled water (dH_2_O) to remove dust and other inessential materials. Then, both clean materials of *H*. *griffithii* were shadow parched under room temperature for two to three weeks continuously and then mature plant material was crushed in an electric pulveriser to obtain a fine concentrate while the immature plant was saved for gemm formulation. The powder was preserved in airtight glass bottles.

### 2.2. Extraction

Accurately weighted mature *H*. *griffithii* (50 g) was macerated with solvent (500 mL) i.e., using a single and binary solvent system (1:1 ratio) comprising of acetone (A), ethanol (E), acetone-ethanol (A-E), methanol (M), acetone-methanol (A-M), methanol-distilled water (M-dW), acetone- distilled water (A-dW), *n*-hexane (Nh), *n*-hexane-ethanol (Nh-E), ethanol-distilled water (e-dW), and distilled water (dW) in an Erlenmeyer flask (1000 mL) for 24 h, and the extraction process was also facilitated by ultra-sonication for 30 min at room temperature. The marc, after straining with muslin cloth, was filtered twice with Whatman No. 1 filter paper and the extracts thus produced were combined to concentrate by evaporation (rotary evaporator, Buchni, Switzerland). The concentrated extract of *H*. *griffithii* was finally dried in a hot air oven (Yamato, Japan) at 50 °C to obtain a final crude extract. % yield of *H*. *griffithii* extract was calculated by using the following, Equation (1):

(1) $$\%\;\mathrm{yield}=\frac{\mathrm{Weight}\;\mathrm{of}\;\mathrm{dried}\;\mathrm{extract}}{\mathrm{Weight}\;\mathrm{of}\;\mathrm{dried}\;\mathrm{powder}}\times 100$$

The stock of 4 mg/mL was prepared in ethanol and ultra-sonicated to obtain clear stock solutions.

### 2.3. Gemmotherapeutic Formulations

The dried immature plant materials (young shoots, leaves and root) were blended with ethanol and glycerine (1:4 ratio) and were left stand for 30 days in a cool shaded environment, shaken well from time to time during this period to facilitate the maceration process. After one month, it was decanted and filtered under constant pressure. It was kept again for forty-eight hours, then filtered twice, resulting in liquid known as souche [14]. The souche/gemm extract was stockpiled in a bottle for further study.

### 2.4. Determination of Phytoconstituents

Phytochemicals of both (gemm and native *H. griffithii*) extracts were estimated by different qualitative as well as quantitative analysis. Alkaloids were detected by following the methods of Verma et al. [15]. Saponins, steroids, flavonoids, tannins, and glycosides were determined adopting standard protocols [16,17]. Both extracts (gemm and native *H*. *griffithii*) (0.1 g) were digested with conc. HNO_3_ (5 mL) and H_2_O_2_ (2 mL) in a digestion flask and diluted with double deionized H_2_O_2_ (upto 10 mL). For complete digestion while sustaining the recovery of volatile components, both samples were heated on a hot plate (MI0102005, Four E’s Scientific, Guangzhou, China) at 105 °C for 4–5 h. The concentration of three elements, namely Mg, Ca, and Fe, was determined by atomic absorption spectrophotometer (AAnalyst 300, Perkin Elmer, Waltham, MA, USA) following the earlier-described procedure [18,19]. The content of Cl^-^ was determined volumetrically, while Na and K were determined by flame photometer (Jenway, PFP-7, Dunmow, UK).

Antioxidant potential of both (native and gemm *H*. *griffithii*) extracts was determined by following antioxidant assays. Total phenolic content (TPC) of both (gemm and native *H. griffithii*) extracts was determined by following the reported methods of Jain, et al. [20], using FC (Follin-Ciocalteu) reagent. Both extracts (100 µg/mL) were mixed with FC reagent (500 µL) then added to 15% Na_2_CO_3_ (2 mL). Both solutions were placed for 2 h at room temperature after adding the volume with d·H_2_O. TPC were quantified by using the calibration curve of gallic acid (standard), while the samples without extracts served as blanks. The results were stated as milligrams per gram of dry matter, measured as gallic acid equivalent (GAE). Total flavonoid contents (TFC) of both (gemm and native *H*. *griffithii*) were assayed by following the reported methods of Pranuthi, et al. [21]. Briefly, 0.5 mL of extracts were mixed with 2.25 mL deionized water. After 5 min, 0.15 mL of 5% NaNO_2_ were added, again after 5 min, 10% AlCl_3_·6H_2_O (0.3 mL) were added and then finally 1 M NaOH (1.0 mL) was added after approximately 5 min at room temperature. OD of reaction mixture was monitored spectrophotometerically at λ_max_ 510 nm. TFC were quantified by using the calibration curve of Quercetin (standard) and results were stated as milligrams per gram of dry matter, measured as quercetin equivalent (QE).

2,2-Diphenyl-1 picrlhudrazyl (DPPH) radical assay was performed to check the free radical scavenging activity of both extracts [22]. Briefly, different concentrations (100–900 μL) of extracts (gemm and native *H*. *griffithii*) were mixed with methanol. Stock solution was prepared by adding 1 mL of extract in 3 mL of methanol solution of DPPH and incubated at room temperature for about 30 min. Optical density was monitored spectrophotometerically at λ_max_ 517 mm. Inhibition of free radicals (%) was calculated using the following, Equation (2):

(2) $$\mathrm{Percent}\;\left(\%\right)\;\mathrm{Inhibition}=\frac{\mathrm{Optical}\;\mathrm{Density}\;\mathrm{of}\;\mathrm{control}-\mathrm{Optical}\;\mathrm{Density}\;\mathrm{of}\;\mathrm{sample}}{\mathrm{Optical}\;\mathrm{Density}\;\mathrm{of}\;\mathrm{Sample}}\times 100$$

### 2.5. Determination of Antimicrobial Activity

Gram-positive bacterial strains included in this study were *Bacillus subtilis* NCTC 10,400, *Bacillus cereus* NCTC 7464, *S*. *aureus* NCTC 6571, Gram-negative *Escherichia coli* ATCC 8739, *P*. *aeruginosa* NCTC 1662, *C*. *albicans* IBL-01, and fungal strains *Candida krusei* ATCC 6258 and *Aspergillus flavus* QC 6158 were obtained from Department of Microbiology, Govt. College University Faisalabad. The inocula of all tested bacterial and fungal strains were prepared following colony suspension method [23]. Colonies formed overnight cultures at 37 °C on nutrient agar (NA, Oxoid)/Potato dextrose agar (PDA, Oxoid), and were picked to prepare test organism suspension in saline solution to provide an OD of approximately 0.1 at 600 nm and diluted by transferring 0.1 mL of suspension to 9.9 mL of sterile nutrient broth/sabouraud agar broth before being used.

#### 2.5.1. Disc Diffusion and Agar Dilution Methods

Sterilized solid growth media in petri dishes (9 cm diameter) were swabbed uniformly with 1 mL of above-mentioned culture media (10^5^–10^6^ CFU/mL) for disk diffusion assay [23]. Sterile paper discs (6 mm in diameter) impregnated with extracts (10 µL) were placed on each solid agar plate by pressing tightly and placed under appropriate cultivation conditions (at 35 °C for 18–24 h for bacterial strains and for 48 h for fungal strains). All experiments were run in triplicate using discs impregnated with ciprofloxacin & fluconazole as reference drug (positive control) and with ethanol serving as negative control. At the end of period, the inhibition zones formed on the media were measured with a transparent ruler in millimetres. The reported method of Afolayan and Meyer, [24] was followed to determine the antimicrobial activity of extracts by agar dilution method, in which different concentrations of extracts (100–10,000 µg/mL) were prepare in nutrient/sabouraud agar at 50 °C. Then, 100 µL of standardized tested microbial cultures were dispensed aseptically and spread uniformly on the agar plates. The agar plates containing 5% ethanol without extracts and blank plates served as negative controls. Each test was performed in triplicate and placed under appropriate cultivation conditions (at 35 °C for 18–24 h for bacterial strains and at 39 °C for 48 h for fungal strains). A test plate having invisible microbial growth is considered as the extract’s MIC (minimum inhibitory concentration).

#### 2.5.2. Determination of Minimum Inhibitory Concentration (MIC) and Minimum Bactericidal Concentration (MBC)

The minimum concentration which maintains or reduces inoculum’s viability, determined by serial dilution method, is known as the minimum inhibitory concentration (MIC). Different concentrations ranging from 9.75–10,000 µL/mL of both extracts and 0.0098–10 µg/mL of ciprofloxacin & fluconazole were differently prepared in sabouraud dextrose/nutrient agar broth by serial dilutions [25]. Around 100 µL of each tested culture strain wasinoculated in each tube while the blank tubes without cultural suspension served as sterility control. MICs were observed after incubating all tubes under appropriate cultivation conditions (at 37 °C for 18–24 h for bacterial strains and at 39 °C for 48 h for fungal strains) and the tube with no visible growth was considered as the MIC. MBC was determined by taking 100 µL aliquot from the first turbid and all clear tubes in the series following the methods of Shanholtzer, et al. [26]. The tubes were slightly mixed by tinting them before sampling. Each aliquot was placed in one streak only in the center on the antibiotic-free medium (nutrient/sabouraud agar) and placed at room temperature till the medium dried (for approximately 30 min), and then tested strains were subcultured with sterile cotton swab. The MBC-determining lawned plates were then incubated at appropriate conditions (at 37 °C for 18–24 h for bacterial strains and at 39 °C for 48 h for fungal strains); the plates exhibiting no visible growth after incubation periods were considered as MBCs for both extracts [27]. These observations were also matched with tubes having no visible microbial growth in MIC test tubes after 48 h.

#### 2.5.3. Evaluation of Antibiosis (Bacteriostatic or Bactericidal) Mechanisms

MBC/MIC ratio or MIC index was calculated to determine whether the extracts are bacteriostatic or bactericidal as described by Shanmughapriya, et al. [28]. The extracts were considered as bactericidal if the value of MBC/MIC ratio was ≤2.0, bacteriostatic if ≥2 but considered as ineffective if ≥16.

#### 2.5.4. Rate of Kill Determination

Rate of microbial killing of both extracts (native and gemm) was determined by modifying the reported methods of Eliopoulos and Moellering, [29]. McCartney bottles having 10 mL of nutrient/sabouraud dextrose broth were incorporated for both extracts at ½ MIC, MIC, and 2MIC while two McCartney bottles, i.e., one without test organisms and other without extracts, served as controls, and approximately 105 CFU/mL dense inoculum was inoculated in all. All bottles were incubated at 37 °C for 18–24 h for bacterial strains and at 39 °C for 48 h for fungal strains in an orbital shaker at 120 rpm and the emergent bacterial colonies counted, CFU/mL calculated, and then compared with controls after incubation.

### 2.6. In-Vitro Hemolytic Activity

Cytotoxic potential of both (native and gemm) extracts was determined by hemolytic assay [30]. Blood samples were collected into heparinized vacuettes through venepuncture from two non-smoker healthy female volunteers after taking informed consent. The tubes were centrifuged at 4 °C for 10 min at 1000× *g* to remove plasma after gentle swirling. The erythrocytes obtained were washed thrice with five volumes of chilled phosphate buffer saline (10 mM PBS consisting of 1.9 mM NaH_2_PO_4_, 8.1 Na_2_HPO_4_ and 150 mM NaCl, pH: 7.4). Erythrocytes (180 µL) were mixed with both extracts (20 µL each) and both mixtures were incubated at 37 °C for 30 min. Mixture was instantly centrifuged after incubation at 1000× *g* for 5 min. Supernatant (100 µL) was taken (discarding pellets) and diluted up to 1 mL by adding 900 µL chilled phosphate buffer saline (pH: 7.4). Optical density was monitored at λ_576_ nm, and ABTS (for complete haemolysis) and phosphate buffer saline (pH: 7.4) were used as positive as well as negative controls respectively. Percent (%) haemolytic activity was calculated using Equation (3).

(3) $$\mathrm{Percent}\;\left(\%\right)\;\mathrm{RBC}\;\mathrm{lysis}=\frac{\mathrm{Optical}\;\mathrm{Density}\;\mathrm{of}\;\mathrm{sample}-\mathrm{Optical}\;\mathrm{Density}\;\mathrm{of}\;\mathrm{control}}{\mathrm{Optical}\;\mathrm{Density}\;\mathrm{of}\;\mathrm{Standard}}\times 100$$

### 2.7. In-Vitro Thrombolytic Activity

Thrombolytic activity in terms of in vitro clot lysis of both (gemm and native *H*. *griffithii*) extracts was evaluated by the methods of Kawsar, et al. [31]. Briefly, 2 mL blood (collected from two healthy female volunteers after informed consent) was poured into pre-weighed Eppendorf tubes and incubated for about 30 min (until a clot formed) at 37 °C. After clot formation, serum was discarded (carefully, to avoid the clot breaking) and the Eppendorf tube was weighed again to determine the weight of the clot. The weight of clot was calculated using the Equation (4).

(4) Weight of clot = Weight of eppendorf tube conatining clot−Weight of empty eppendorf tube

A volume of 100 µL of both extracts (10 mg/mL) was added to Eppendorf tubes containing pre-weighed clots. Two other Eppendorf tubes, in which one held Streptokinase (100 µL) and other held distilled H_2_O (100 µL), were used as a standard and negative control, respectively. All Eppendorf tubes were then incubated for about 3 h at 37 °C and clot lyses were observed. After the completion of the incubation period the fluids formed were discarded and remaining weight was determined to check the clot’s lyses percentage. Percent (%) clot lysis was determined by the following formulae:

(5) $$\mathrm{Percent}\;\left(\%\right)\;\mathrm{clot}\;\mathrm{lysis}=\frac{\mathrm{Final}\;\mathrm{Weight}}{\mathrm{Clot}\;\mathrm{Weight}}\times 100$$

### 2.8. Data Analysis

Data was analyzed using Excel spread sheets, Microsoft version 2010. Results were expressed as Standard Deviation (SD) and ± Mean values.

## 3. Results and Discussion

**Figure 1:**
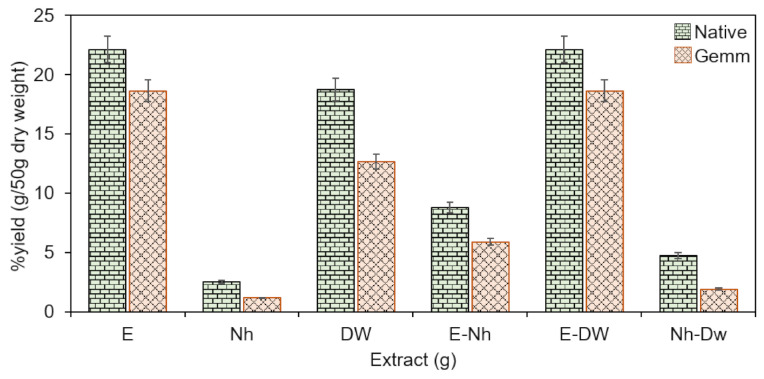
Percent (%) yield of both Native and gemm extracts of *H. griffithi* using mono and binary solvents (1:1) for extraction. E: ethanol, Nh: *n*-hexane, DW, distilled water.

The percent yield of both extracts (native and gemm extracts of *H*. *griffithii*) recovered by employing three different solvents and their combination via ultra-sonication and maceration processes are described in the Figure 1. Different trends in extract yield were achieved by keeping extraction process and starting mass constant with single solvent or their combinations. Results indicated that maximum extract yields for both gemm and native extracts, 18.65 ± 1.85, 22.12 ± 0.50 and 18.65 ± 0.20, 22.12 ± 0.85% (*w/w*), were obtained in E-Dw and E solvents while minimum extract yields 2.5 ± 0.40, 1.16 ± 0.25 and 4.75 ± 0.45, 5.89 ± 0.60% (*w/w*) were obtained with Nh-Dw and Nh respectively. It can be suggested from these results that different solvents systems behave differently to pick up the components and a wide range of polarity is employed wisely for maximum extraction. It was also observed that polar solvents presented maximum extraction efficacy compared to non-polar ones. Therefore, for extract recovery, choice of solvent is a critical factor [17]. Qualitative as well as quantitative analysis showed that all the essential, potentially active, and medicinally important principles are present in both forms of *H*. *griffithii* (Table 1). Results indicated that levels of alkaloids, flavonoids, saponins and phenolics were higher in gemm extracts of *H*. *griffithii* than native extracts. Glycosides appeared to be abundantly present phytoconstituents in the native extract followed by < saponins < tannins < phenolics < flavonoids < alkaloids while phenolics are present abundantly followed by flavonoids < saponins < glycosides < tannins < alkaloids in gemm extracts (Table 1). High values of constituents in the gemm extract may from growing shoots, roots and leaves that are rich in these phytochemicals.

**Figure 2:**
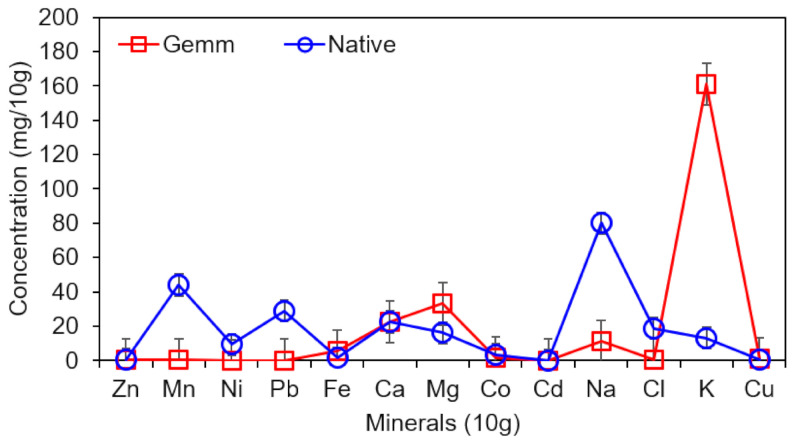
Mineral profile of both Native and gemm extracts of *H. griffithi*.

From the results (Table 1), it can be postulated that the age of plants and seasonal variation affect the phytoconstituents of the plants. It is most probable that such phytochemical components may vary in levels in hot summer days and in flowering season. Comparison between two forms indicate that gemm extract of *H*. *griffithii* has a higher concentration of affective ingredients than the native form. As remedies made of developing tissues, young shoots and roots are true concentrates of plants’ energy and validity. This explains the wide range of applications and effectiveness of gemmo remedies [32]. Mineral contents (both macro-nutrients i.e., Ca, K, Na and Fe and micro-nutrients i.e., Zn, Mn, Ni, Pb, Cl, etc.) of both extracts (native and gemm) of *H*. *griffithii* are depicted in Figure 2. Predominately, macro-nutrients varied considerably between both forms of *H*. *griffithii*. Native extract had the highest Na content (80 mg/10 g) and lowest Cu^+^ contents (0.18 mg/10 g) while gemm extract was found to be a rich source of K content (161 mg/10 g) with lowest content of Ni (0.044 mg/10 g). Both extracts of *H*. *griffithii* had equal contents of Zn and Ca (0.54 mg/10 g and 22.36 mg/10 g respectively). It can be observed that gemm extract is a rich source of K, Ca, Mg and Fe whereas the contents of other minerals such as Na, Ni, and Pb were significantly high in native extract. Results indicated that gemm extract is a rich source of K. Relatively, contents of Mg, Ca and Fe were lower by 5-, 7- and 30-fold, respectively, than K content in gemm extract of *H*. *griffithii*. The decreasing order of K > Fe > Ca > Mg is in accordance with the findings of Ahmad, et al. [13]. Results reveal that the diuretic effect is attributed due to high contents of K. It has been reported that K has ability to reduce elevated blood pressure by attenuating vascular contraction [33]. The diet of the populations in developing countries is mainly composed of vegetables, starchy foods, eggs, and meat. Due to the presence of starchy foods in the majority of meals, many of them are suffering from nutrient deficiency; therefore, nutripharm formulations are of great interest [34]. Our results indicate that the gemmotherapeutic formulation of *H*. *griffithii* is much more appropriate for obtaining these micro- and macronutrients, as well as phytochemicals, than native forms.

### 3.1. Antimicrobial Activity

The ability of microorganism (bacteria and fungi) to exhibit palpable growth under the influence of both extracts of *H*. *griffithii* (native and gemm extracts) and standard antibiotics was tested by disk diffusion and agar dilution methods. The MIC values as ascertained by disk-diffusion agar method were aligned predominately from 20.86 ± 0.020 to 38.75 ± 1.00 µg/mL for Gram-positive bacteria (Table 2), from 12.30 ± 0.45 to 20.28 ± 0.75 µg/mL for Gram-negative bacteria (Table 3) and from 5.80 to 21.45 75 µg/mL for fungus (Table 4), revealing *A*. *flavus* as the most susceptible organism while *B*. *cereus* was resistant as compared to other bacterial species because its growth was inhibited in the range of 36.40 ± 0.65 to 38.75 ± 0.65 µg/mL. MIC values of both extracts of *H*. *griffithii* (native and gemm extracts) were determined by agar dilution method which resulted in complete growth inhibition of all microbial species under investigation.

Generally, irrespective of the extract, MIC values obtained by agar dilution method were 2–20 times lower than the disk diffusion method. Native extract was much less efficient than gemm extract against all tested microbial species because tested *H*. *griffithii* extracts (native and gemm) differ consequently in their phenolic contents. However, both extracts exhibited antimicrobial potential, yet the response for each tested microbial sp. was different. MIC values of standard antibiotics, ciprofloxacin against selected bacterial species were mostly below 35 µg/mL and MBC values ranged within 10 µg/mL and 45 µg/mL with the exception of *S*. *aureus* NCTC 6571 having 38.75 ± 0.90 µg/mL MIC and 76.50 ± 0.25 µg/mL MBC values, whereas MIC values of fluconazole against selected species were generally below 20.01 ± 0.75 µg/mL with MBC values below 1.1 µg/mL except *A*. *flavus* QC 6158 with 38.85 ± 1.45 MIC and 1.7 ± 1.13 µg/mL MBC values. MIC values of native extract against selected microbial sp. ranged between 5.80 ± 1.00 µg/mL and 22.25 ± 0.045 µg/mL and MBCs were generally below 52.30 ± 1.00 with the exception of *B*. *cereus* NCTC 7464 (with 38.75 ± 0.65 µg/mL MIC and 77.5 ± 0.40 µg/mL MBC) while MICs of gemm extract were generally below 20.86 ± 0.02 µg/mL with 24.00 ± 1.88 µg/mL MBC with highest MIC and MBC (36.40 ± 1.00 µg/mL and 38. 20 ± 0.80 µg/mL) against *B*. *cereus* NCTC 7464 (Table 5) while MIC values of gemm extract (≤26.40 ± 1.00 µg/mL) were generally below the MICs of native. The mechanism of antibiosis showed that both extracts of *H*. *griffithii* (native and gemm) along with antibiotics (ciprofloxacin and fluconazole) were highly bactericidal as well as fungicidal even though native extract was evaluated as not as effective as gemm extract. MCB values of both extracts (gemm and native) were two-fold higher than MIC values, similar to standard antibiotics whose MBC values were two to four times higher than their MIC values (Table 5).

The variation in values of MIC and MBC proposed a selective antimicrobial potential of both extracts, while the diverse susceptibility of varied microbial isolate was extract-concentration dependent. The crude extracts having values of MIC < 1000 µg/mL are considered as active crude extracts [35]. Lower values of MIC indicated higher efficacy while in routine, all phytochemicals having MIC values in range of 100–1000 µg/mL in different susceptibility tests are classified as antimicrobials [36], while the values of MIC and MBC < 1000 µg/mL in the present study were of excellent activity. The values of MIC_index_ ≤ 2 represented bactericidal or fungicidal attributes of native as well as gemm extracts and assumed that bactericidal or fungicidal effects of both extracts could be supposed on most of the tested microorganisms in diseased states also [37]. Equivalent MIC and MBC values indicated high therapeutic potential of both extracts against broad spectrum microbial species. Results in terms of log_10_ CFU/mL changes in viable colonies of selected microbial species in time kill assay indicated that both extracts possessed significant bactericidal/fungicidal activity. Reduction greater or equal to 3 log_10_ CFU/mL in viable colony count relative to initial inoculum is defined as bactericidal or fungicidal activity [37]. The results of in vitro time kill assay of native extract of *H. griffithi* are summarized in Table 6. Table 7 presented the results of in vitro time kill assay of gemm extract of *H. griffithi*. The average log reduction in viable cell counts by incubating microbial species with 1 *×* MICs and 2 *×* MICs were ranged between −1.264 ± 0.50 log_10_ and 1.826 ± 0.80 log_10_ CFU/mL for native while −0.390 ± 0.00 log_10_ and 0.026 ± 0.008 log_10_ CFU/mL for gemm extract. The average log reduction in viable cell count after 8 h incubation was ranged between −4.062 ± 1.008 log_10_ to 1.830 ± 0.00 log_10_ CFU/mL for native whereas −2.875 ± 0.50 log_10_ to −0.922 ± 0.06 log_10_ CFU/mL for gemm extract. The average log reduction in viable cell count after 4 h incubation at 1 *×* MICs was ranged between 1.826 ± 0.80 log_10_ to 2.410 ± 1.55 log_10_ CFU/mL for native and −0.82 ± 0.085 log_10_ to 0.735 ± 0.035 log_10_ CFU/mL while at 8 h reduction ranged between 0.788 ± 0.00 log_10_ to 1.830 ± 0.00 log_10_ CFU/mL for native and −0.122 ± 0.00 log_10_ to −0.922 ± 0.06 log_10_ CFU/mL for gemm extract. The average log reduction in viable cell count after 4 h incubation at 2 *×* MICs was ranged −0.162 ± 0.20 log_10_ to 1.559 ± 0.08 log_10_ CFU/mL for native, −0.390 ± 0.00 log_10_ to 0.120 ± 0.020 log_10_ CFU/mL for gemm extract while −0.121 ± 0.80 log_10_ to −4.062 ± 1.008 log_10_ CFU/mL for native, −0.891 ± 0.80 log_10_ to −2.875 ± 0.50 log_10_ CFU/mL for gemm extract after 8 h incubation.

The results indicated that *A*. *flavus* QC 6158, *C*. *albicans* IBL-01, and *P*. *aeruginosa* NCTC 1662 highly affected microbial sp. by native extract while *C*. *krusei* ATCC 6285, *P*. *aeruginosa* NCTC 1662, *C*. *albicans* IBL-01 and *A*. *flavus* QC 6158 are the most affected microbial sp. by gemm extract of *H*. *griffithii*. The comparison of antimicrobial potential between gemm and native extract of *H*. *griffithii* indicate that at the same concentration gemm extract is more effective, proving that in the growth-stage plant can be found a rich source of active phytochemicals or regulators which are found in very low concentrations in the mature plant. Gemm-remedies are used in low homeo-therapeutically prepared potencies [8]. The bactericidal effect of gemm extract is due to high concentration of phenolics which cause the disruption of microbial plasma membrane via H^+^ donation and intracellular cytosolic low pH via hyper-acidification inhibiting the H^+^-ATPase vital for ATP production [38].

### 3.2. Antioxidant Activity Evaluation

#### 3.2.1. Total Flavonoid Content (TFC)

**Figure 3:**
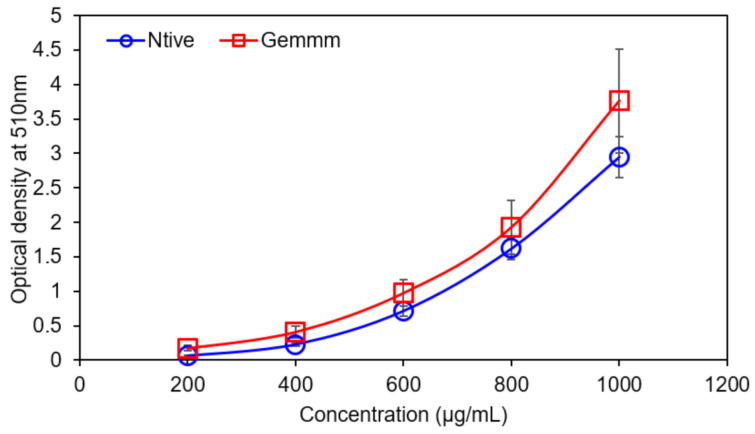
Total Flavonoid Contents of Native and gemm extracts of *H. griffithi*.

Total flavonoid contents were expressed by the quercetin equivalent (QE). Appearance of yellow color indicated the presence of flavonoids, and both forms of *H*. *griffithii* (native and gemm extracts) possessed 21.32 ± 1.06 µg and 43.12 ± 2.45 µg QE/g dry weight of TFC respectively. The comparison between both extracts indicated that highest TFC contents were found in gemm extract (Figure 3). The results indicated that optical density increased gradually with increase in concentration and the highest optical density (3.5) was recorded with 1000 µg/mL gemm extract [39].

**Figure 4:**
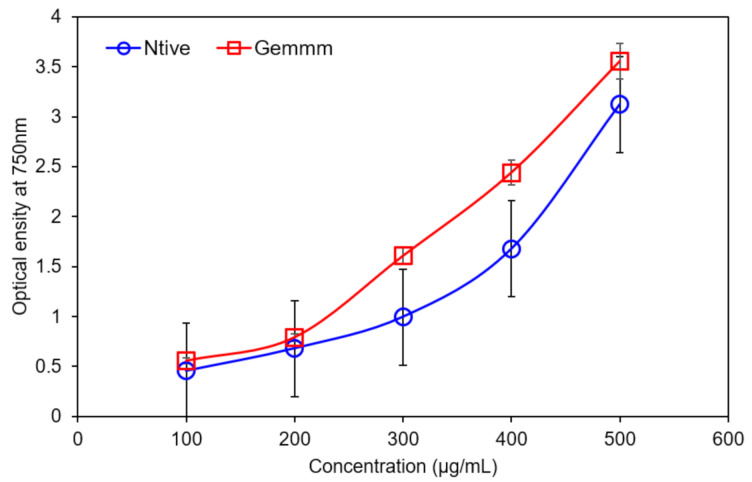
Total Phenolic Contents of Native and gemm extracts of *H. griffithi*.

TPC was determined by the gallic acid equivalent (GA). Appearance of red colour indicates the presence of flavonoids and both extracts *H*. *griffithii* (native and gemm extracts) have variation in the TPC. Maximum phenolic contents 97 mg/g were found in gemm extracts. The comparison between both *H*. *griffithii* (native and gemm extracts) indicated that gemm forms rich source of TPC (Figure 4). Both TPC and TFC have antioxidant potential due to their stable radical intermediates and e^-^ donating ability of H_2_ [22].

The HPLC profile *H*. *griffithii* gemm extract indicated that gemm extract contained higher amounts of phenolics. Results indicated that gallic acid with R_t_ = 2.66 min, p.coumeric acid with R_t_ = 5.44 min, catechin with R_t_ = 3.41 min, vanillin with R_t_ = 4.00, ellagic acid with R_t_ = 4.12 min, salicylic acid with R_t_ = 19.81 (Figure S1). The concentration of identified phenolics ranged from 650 to 6000 mg/g in gemm extract while 480–2250 mg/g in native extract. The results of qualitative analysis revealed significant variation of contents in both extracts of *H*. *griffithii*. Numerous environmental factors such as rainfall, climate, altitude etc., influence significantly on the production of different phytochemicals [13]. Phenolics, due to antioxidant potential, are correlated with a lower threat of mortality from numerous diseases such as acute hypertension, cardiovascular diseases, diabetes, and various cancers [33].

#### 3.2.2. DPPH Radical Scavenging Assay

**Figure 5:**
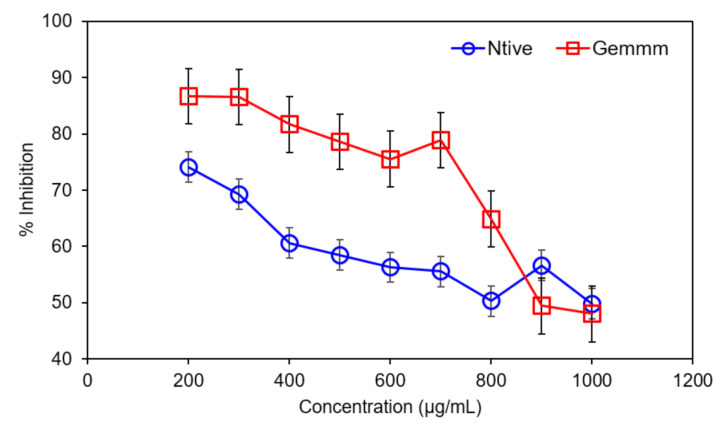
DPPH radical scavenging activity of Native and gemm extracts of *H. griffithi*.

The DPPH (1,1-diphenyl-2-picryl-hydrazyl) was very significant and an appropriate way to determine the antioxidant potential of both extracts of *H*. *griffithii* (native and gemm extracts). DPPH radical, visible by a purple color, is expressed due to the presence of odd electrons; these electrons form antioxidant complexes and cause decolorization which was measured by changing the absorbance of DPPH [12]. Gemm extracts exhibited higher % inhibition (91.61 ± 5.2%) than native extract. DPPH of both forms of *H*. *griffithii* are summarized in Figure 5. The results revealed that the higher the values of TPC and TFC, the higher was the free radical scavenging or % inhibition, and the greater was the antioxidant potential [40]. Hence, the results revealed *H*. *griffithii* has the richest source of antioxidants of all other medicinal plants; moreover, gemm formulations are the best source to fulfil the demands of an increasing population.

### 3.3. Thrombolytic Activity of Haloxylon Griffithii

**Figure 6:**
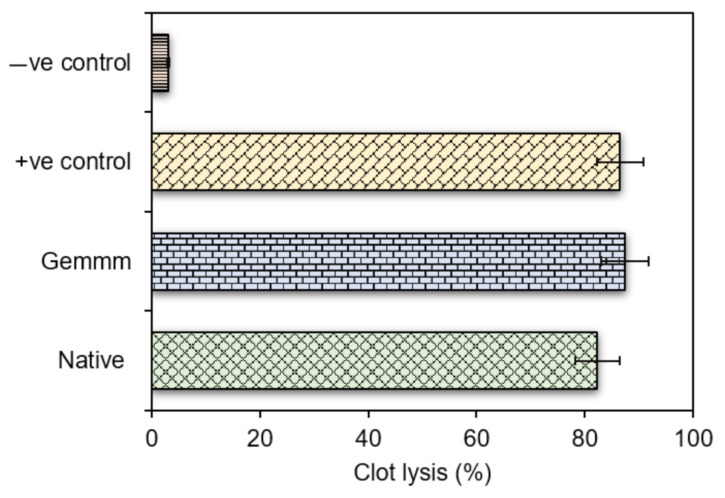
Percent (%) of thrombolysis of gemm and native extracts of *H. griffithi*.

The thrombolytic activity of both extracts of *H*. *griffithii* (native and gemm extracts) was determined by % clot lysis and both forms showed significant clot lysis. Significant differences in % clot lysis between positive and negative controls were observed. The results of thrombolytic activity showed that streptokinase (positive controls) causes 86.5 ± 1.01% clot lysis while ethanol (negative control) causes negligible clot lysis (3.06 ± 1.88%), clearly indicating that ethanol did not influence the dissolution of clots. Highest % clot lysis was observed with gemm extracts (87.9 ± 0.85% clot lysis). The % clot lysis obtained after clot treatment with controls and both extracts is exhibited in Figure 6. Literature reported number of thrombolytic drugs with stark side effects that may sometimes cause embolism and bleeding [41]. In the present study, gemm formulation of *H*. *griffithii* proved thrombolytic activity with high efficacy and safety as compared to controls. Secondary metabolites such as saponins and flavonoids are the rich source of thrombolytic activity [42]. Both studied extractives of *H*. *griffithii* (native and gemm extracts) have high concentration of these metabolites which degraded the fibrin clot, which is the one possible mechanism of its thrombolytic potential. However, there are reports that thrombolytic activity and antibacterial activities of medicinal plants are directly related [43].

### 3.4. Determination of Cytotoxicity

**Figure 7:**
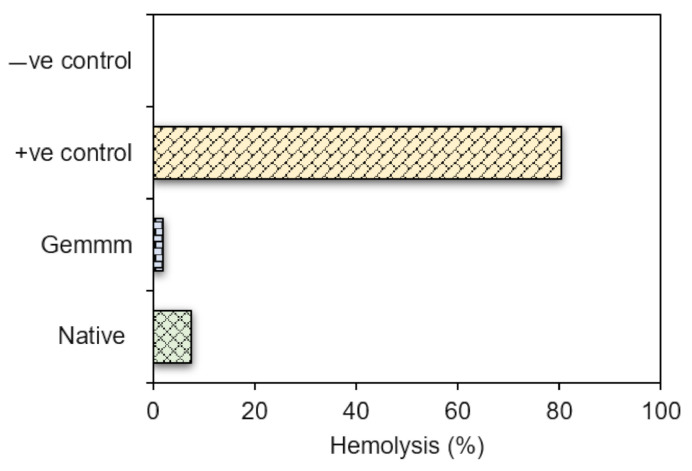
Hemolytic activity of Native and gemm extracts of *H. griffithi*.

In vitro cytotoxic potential of both extracts of *H*. *griffithii* (native and gemm extracts) was determined by haemolytic assay. Lowest haemolytic activity (1.85 ± 0.68%) was observed by gemm extracts. Highest % haemolysis was observed by native extracts (7.58 ± 1.02) while 80.3 ± 8.64% of Triton x-100 (positive control) and 0% haemolysis was shown by normal saline (negative control). Lower haemolytic potential of both extracts (Figure 7) makes them suitable for optimization in pharmaceutical formulation. Both extracts possessing negligible haemolytic activity indicate they are non-toxic in nature.

## 4. Conclusions

In the present study, we demonstrated for the first time the chemical composition and antimicrobial, haemolytic, thrombolytic, and radical scavenging potential of gemmotherapeutic formulations as well as native extract of *H*. *griffithii*. Gemm extract of *H*. *griffithii* showed better antimicrobial activity against Gram-positive (MIC = 12.30 ± 0.45 µg/mL against *S*. *aureus* NCTC 6571) and Gram-negative bacteria (13.85 ± 1.20 against *P*. *aeruginosa* NCTC 1662) and fungi (6.75 ± 0.08 against *C*. *albicans* IBL-01), demonstrating antioxidant and thrombolytic potential with negligible cytotoxic properties at same concentration as native extract. These findings will aid in the exploration for novel phytomedicines or their active constituents, employed as remedies for cardiovascular and infectious diseases. Therefore, the present work is assumed to be a beginning point to adopt the gemmotherapeutic formulations of natural flora from the Baluchistan plains of Pakistan on the treatment of infectious and cardiovascular diseases, instead of broad-spectrum antibiotics.

## Figures and Tables

**Table 1:** Extraction and quantification of phytochemicals in native and gemm extracts of *H*. *griffithi*.

Sr. No.	Constituents	Native *H*. *Griffithi*		Gemm *H*. *Griffithi*	
		Mass (g)	%Yield	Mass (g)	%Yield
1	Alkaloids	0.28	2.8	0.48	4.8
2	Saponins	1.30	13	2.00	20
3	Tannins	1.20	12	0.70	7
4	Glycosides	2.40	24	1.70	17
5	Flavonoids	0.841	8.41	2.46	24.6
6	Phenolics	0.880	8.80	2.97	29.7

**Table 2:** Antimicrobial activity of native and gemm extracts of *H*. *griffithi* expressed as MIC (µg/mL) determined by disk diffusion and agar dilution methods against Gram-positive bacterial strains.

Plant Extracts	*B*. *subtilis* NCTC 10,400		*B*. *cereus* NCTC 7464		*S*. *aureus* NCTC 6571	
	Disk Diffusion	Agar Dilution	Disk Diffusion	Agar Dilution	Disk Diffusion	Agar Dilution
Native Extract	22.25 ± 0.045	5.60 ± 1.20	38.75 ± 0.65	7.98 ± 0.35	14.75 ± 0.25	2.50 ± 0.20
Gemm Extract	20.86 ± 0.020	5.5 ± 0.80	26.40 ± 1.00	6.68 ± 0.08	12.30 ± 0.45	4.60 ± 0.70
Ciprofloxacin	10.50 ± 0.00	1.25 ± 0.75	28.80 ± 0.55	26.75 ± 0.70	38.75 ± 0.90	18.25 ± 0.45
Fluconazole	ND	0.025 ± 0.00	ND	0.154 ± 0.00	ND	0.033 ± 0.00
Ethanol	ND	0.033 ± 0.00	ND	0.045 ± 0.00	ND	0.021 ± 0.00

**Table 3:** Antimicrobial activity of native and gemm extracts of *H*. *griffithi* expressed as MIC (µg/mL) determined by disk diffusion and agar dilution methods against Gram-negative bacterial strains.

Plant Extracts	*E. coli* ATCC 8739		*P*. *aeruginosa* NCTC 1662	
	Disk Diffusion	Agar Dilution	Disk Diffusion	Agar Dilution
Native Extract	20.28 ± 0.75	5.25 ± 1.20	18.80 ± 0.25	2.1 ± 0.06
Gemm Extract	18.15 ± 0.50	4.90 ± 0.80	13.85 ± 1.20	1.89 ± 0.05
Ciprofloxacin	34.45 ± 0.00	5.68 ± 0.40	26.15 ± 0.15	1.42 ± 0.20
Fluconazole	ND	0.078 ± 0.00	ND	0.073 ± 0.00
Ethanol	ND	0.156 ± 0.00	ND	0.025 ± 0.00

**Table 4:** Antimicrobial activity of native and gemm extracts of *H*. *griffithi* expressed as MIC (µg/mL) determined by disk diffusion and agar dilution methods against fungal strains.

Plant Extracts	*C*. *krusei* ATCC 6285		*A*. *flavus* QC 6158		*C*. *albicans* IBL-01	
	Disk Diffusion	Agar Dilution	Disk Diffusion	Agar Dilution	Disk Diffusion	Agar Dilution
Native Extract	21.45 ± 0.50	10.22 ± 0.02	5.80 ± 1.00	0.65 ± 0.00	12.00 ± 0.25	0.95 ± 0.00
Gemm Extract	8.9 ± 1.00	6.95 ± 0.55	5.80 ± 0.080	0.78 ± 0.00	6.75 ± 0.08	1.25 ± 0.45
Ciprofloxacin	ND	0.12 ± 0.00	ND	0.015 ± 0.00	ND	0.76 ± 0.15
Fluconazole	17.46 ± 0.85	3.25 ± 1.008	22.50 ± 0.82	5.80 ± 1.25	20.05 ± 0.75	5.80 ± 0.02
Ethanol	ND	ND	ND	0.052 ± 0.00	ND	0.033 ± 0.00

**Table 5:** Antimicrobial activity of native and gemm extracts of *H*. *griffithi*.

Tested Microbial Species	MIC	MBC	MIC_index_
**Ciprofloxacin**			
*B. subtilis* NCTC 10,400	10.50 ± 0.15	12.50 ± 0.00	1.2 ± 0.075
*B. cereus* NCTC 7464	28.80 ± 0.55	30.08 ± 0.10	1.07 ± 0.32
*S. auresus* NCTC 6571	38.75 ± 0.90	76.50 ± 0.25	2.00 ± 0.57
*E. coli* ATCC 8739	34.45 ± 0.00	44.00 ± 1.25	1.2 ± 0.62
*P. aeruginosa* ATCC 6285	26.15 ± 0.15	26.20 ± 0.20	1.0 ± 0.17
*C. krusei* ATCC 6285	ND	ND	ND
*A. flavus* QC 6158	ND	ND	ND
*C. albicans* IBL-01	ND	ND	ND
**Fluconazole**			
*B. subtilis* NCTC 10,400	ND	ND	ND
*B. cereus* NCTC 7464	ND	ND	ND
*S. auresus* NCTC 6571	ND	ND	ND
*E. coli* ATCC 8739	ND	ND	ND
*P. aeruginosa* ATCC 6285	ND	ND	ND
*C. krusei* ATCC 6285	17.46 ± 0.85	20.45 ± 0.50	1.1 ± 0.67
*A. flavus* QC 6158	22.50 ± 0.82	38.85 ± 1.45	1.7 ± 1.13
*C. albicans* IBL-01	20.01 ± 0.75	20.05 ± 0.28	1.00 ± 0.14
**Native Extract**			
*B. subtilis* NCTC 10,400	22.25 ± 0.045	44.25 ± 0.42	2.00 ± 0.23
*B. cereus* NCTC 7464	38.75 ± 0.65	77.5 ± 0.40	2 ± 0.52
*S. auresus* NCTC 6571	14.75 ± 1.00	14.70 ± 0.35	1.00 ± 0.67
*E. coli* ATCC 8739	20.28 ± 0.75	52.30 ± 1.00	2.00 ± 0.87
*P. aeruginosa* ATCC 6285	18.80 ± 0.25	18.90 ± 0.25	1.00 ± 0.25
*C. krusei* ATCC 6285	21.45 ± 0.50	21.38 ± 0.08	1.00 ± 0.29
*A. flavus* QC 6158	5.80 ± 1.00	10.75 ± 0.20	2.00 ± 0.60
*C. albicans* IBL-01	12.00 ± 0.25	24.25 ± 0.00	2.25 ± 0.25
**Gemm Extract**			
*B. subtilis* NCTC 10,400	20.86 ± 0.02	20.50 ± 0.88	1.01 ± 0.45
*B. cereus *NCTC 7464	26.40 ± 1.00	28.20 ± 0.80	1.04 ± 0.90
*S. auresus* NCTC 6571	12.30 ± 0.45	24.00 ± 1.88	1.95 ± 1.17
*E. coli* ATCC 8739	18.15 ± 0.50	18.50 ± 0.95	1.01 ± 0.72
*P. aeruginosa* ATCC 6285	13.85 ± 1.20	13.50 ± 0.50	1.00 ± 0.85
*C. krusei* ATCC 6285	8.0 ± 1.00	8.25 ± 0.02	1.03 ± 0.51
*A. flavus* QC 6158	5.80 ± 1.00	10.98 ± 0.00	1.89 ± 0.50
*C. albicans* IBL-01	6.75 ± 0.25	8.75 ± 0.00	1.30 ± 0.12

**Table 6:** In vitro time kill assay of native extract of *H*. *griffithi*.

Tested Microbial Species	Log_10_ Kill ½ × (MIC)			Log_10_ Kill 1 × (MIC)			Log_10_ Kill 2 × (MIC)		
	0 h	4 h	8 h	0 h	4 h	8 h	0 h	4 h	8 h
*B*. *subtilis*	3.187 ± 0.00	3.480 ± 0.90	4.233 ± 1.00	3.207 ± 0.15	2.135 ± 0.10	1.183 ± 0.90	3.240 ± 0.80	−1.264 ± 0.50	−3.324 ± 0.20
*B*. *cereus*	3.229 ± 0.02	4.122 ± 0.45	4.899 ± 0.40	3.248 ± 0.20	2.410 ± 1.55	1.830 ± 0.25	2.125 ± 0.50	1.298 ± 0.75	−2.517 ± 0.35
*S*. *auresus*	3.285 ± 1.00	4.242 ± 0.50	5.442 ± 0.25	3.316 ± 1.20	2.158 ± 0.25	1.254 ± 1.25	3.401 ± 1.50	1.264 ± 0.50	−2.412 ± 0.50
*E. coli*	3.270 ± 0.25	3.820 ± 0.70	5.416 ± 0.0	3.410 ± 1.25	2.258 ± 0.24	1.266 ± 0.45	3.164 ± 0.30	1.559 ± 0.08	−4.062 ± 1.008
*P*. *aeruginosa*	4.150 ± 0.25	5.283 ± 0.25	4.262 ± 0.05	3.442 ± 0.80	2.146 ± 0.50	1.045 ± 0.20	2.297 ± 0.45	−0.162 ± 0.20	−0.150 ± 0.60
*C*. *krusei*	2.953 ± 0.65	3.054 ± 0.85	6.210 ± 0.80	3.668 ± 0.25	1.826 ± 0.80	1.283 ± 0.05	4.346 ± 0.50	1.980 ± 0.00	−1.819 ± 0.09
*A*. *flavus*	1.690 ± 0.95	2.755 ± 1.15	5.896 ± 0.00	2.688 ± 0.55	1.929 ± 0.75	1.231 ± 0.15	4.349 ± 0.25	−0.475 ± 0.00	−2.464 ± 0.50
*C*. *albicans*	1.285 ± 0.45	3.272 ± 0.85	4.168 ± 0.50	3.435 ± 0.60	2.122 ± 0.75	0.788 ± 0.28	−1.216 ± 0.20	−0.425 ± 0.00	−0.121 ± 0.80

**Table 7:** In vitro time kill assay of gemm extract of *H*. *griffithi*.

Tested Microbial Species	Log_10_ Kill ½ × (MIC)			Log_10_ Kill 1 × (MIC)			Log_10_ Kill 2 × (MIC)		
	0 h	4 h	8 h	0 h	4 h	8 h	0 h	4 h	8 h
*B*. *subtilis*	1.187 ± 0.089	1.895 ± 1.25	2.102 ± 0.92	1.208 ± 1.50	0.122 ± 0.008	−0.817 ± 1.20	1.222 ± 0.008	−0.736 ± 0.15	−1.213 ± 0.25
*B*. *cereus*	1.20 ± 0.035	2.123 ± 1.09	2.854 ± 0.02	1.164 ± 0.03	0.399 ± 0.75	−0.812 ± 0.00	1.310 ± 0.78	−0.702 ± 0.20	−0.526 ± 1.25
*S*. *auresus*	1.288 ± 0.010	2.343 ± 0.25	3.316 ± 1.05	1.211 ± 0.45	0.142 ± 1.25	−0.128 ± 0.00	1.210 ± 1.00	−0.541 ± 0.45	−0.502 ± 0.75
*E. coli*	1.191 ± 0.950	1.510 ± 0.08	3.314 ± 1.50	1.218 ± 0.50	0.143 ± 1.00	−0.735 ± 0.05	1.241 ± 0.25	−0.839 ± 0.50	−2.875 ± 0.50
*P*. *aeruginosa*	2.129 ± 0.250	3.384 ± 0.98	1.198 ± 1.008	1.212 ± 0.75	0.026 ± 0.008	−0.922 ± 0.06	0.246 ± 0.035	0.120 ± 0.020	−1.543 ± 1.45
*C*. *krusei*	3.450 ± 0.008	3.743 ± 1.25	4.480 ± 0.75	1.427 ± 1.00	−0.82 ± 0.085	−0.620 ± 0.25	0.411 ± 0.02	−0.390 ± 0.00	−0.891 ± 0.80
*A*. *flavus*	3.442 ± 0.093	4.322 ± 1.00	4.659 ± 0.25	1.642 ± 0.09	−0.84 ± 1.45	−0.619 ± 0.05	0.124 ± 0.008	−0.525 ± 0.45	−0.1343 ± 0.20
*C*. *albicans*	2.399 ± 0.76	3.512 ± 0.02	4.736 ± 1.00	1.422 ± 0.90	0.735 ± 0.035	−0.122 ± 0.08	−0.220 ± 0.75	−0.575 ± 0.08	−0.879 ± 1.00

## Data Availability

All data belongs to this work is reported herein. HPLC related data is given in the supplementary material (Figure S1). No additional data is associated to this study.

## Supplementary Materials

The following are available online at https://www.mdpi.com/article/10.3390/plants10071427/s1, Figure S1 High-performance liquid chromatography (HPLC) profile *H. griffithii* gemm extract.
