# Supplementary material for: Biological Activities of In-House Developed Haloxylon griffithii Plant Extract Formulations
Source: Plants (Basel). 2021 Jul 13;10(7):1427. doi: 10.3390/plants10071427 (PMC8309412; doi:10.3390/plants10071427)
Supplement: Supplementary file 1 [file plants-10-01427-s001.zip › plants-1229745-supplementary.pdf]

Supplementary material

Research Article

Biological Activities of In-house Developed *Haloxylon griffithii* Plant Extract Formulations

Shagufta Kamal [1,*], Ismat Bibi [2], Kanwal Rehman [3], Ameer Fawad Zahoor [4], Amna Kamal [5], Fatima Aslam [1], Fatmah Ali Alasmary [6], Tahani Mazyad Almutairi [6], Hassna Mohammed Alhajri [6], Siham A. Alissa [7], and Hafiz M.N. Iqbal [8,*]

[1]Department of Biochemistry, Government College University, Faisalabad 38000, Pakistan; shaguftakamal81@gmail.com (S.K.); Fateemahchaudhary@gmail.com (F.A.).
[2]Department of Chemistry, The Islamia University of Bahawalpur, Bahawalpur 63100, Pakistan; drismat@iub.edu.pk (I.B.).
[3]Department of Pharmacy, University of Agriculture, Faisalabad, Pakistan; kanwalrehman@uaf.edu.pk (K.R.).
[4]Department of Chemistry, Govt. College University, Faisalabad 38000, Pakistan; fawad.zahooe@gmail.com (A.F.Z.).
[5]Department of Chemistry, University of Agriculture, Faisalabad, Pakistan; amina89@gmail.com (A.K.).
[6]Chemistry Department, College of Science, King Saud University, P.O. Box 2455, Riyadh 11451, Saudi Arabia; fasmari@ksu.edu.sa (F.A.A.); talmutari1@ksu.edu.sa (T.M.A.); 441203417@student.ksu.edu.sa (H.M.A.).
[7]Department of Chemistry, College of Science, Princess Nourah bint Abdulrahman University, Riyadh 11671, Saudi Arabia; saalissa@pnu.edu.sa (S.A.A.).
[8]Tecnologico de Monterrey, School of Engineering and Sciences, Monterrey, 64849, Mexico; hafiz.iqbal@tec.mx (H.M.N.I.).
*Correspondence: shaguftakamal81@gmail.com (S.K.); hafiz.iqbal@tec.mx (H.M.N.I.).

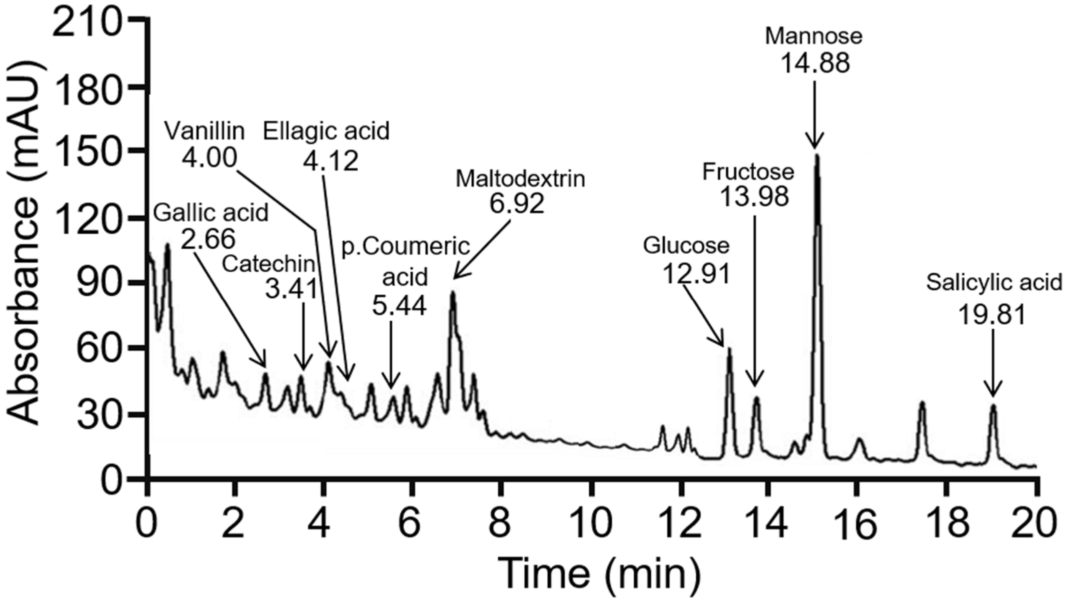

Figure S1. High-performance liquid chromatography (HPLC) profile *H. griffithii* gemm extract.
